# SPIROMETRY IN SCHOOLCHILDREN FOR FIELD STUDIES: DOES TESTING ON DIFFERENT DAYS CHANGE THE RESULT OF THE EXAM?

**DOI:** 10.1590/1984-0462/;2018;36;1;00014

**Published:** 2017-11-13

**Authors:** Janaina Cristina Scalco, Rafaela Coelho Minsky, Camila Isabel Santos Schivinski

**Affiliations:** a Universidade do Estado de Santa Catarina, Florianópolis, SC, Brasil.

**Keywords:** Spirometry, Children, Reproducibility of results, Espirometria, Crianças, Reprodutibilidade dos testes

## Abstract

**Objective:**

To analyze if there is variation in the results of spirometry performed by healthy schoolchildren on different days.

**Methods:**

Cross-sectional study with healthy schoolchildren aged 7 to 12 years old, in Florianópolis, Santa Catarina, Southern Brazil. Prepubescent children, non-athletes, born at full term, without cardiorespiratory, rheumatic, musculoskeletal, neurological and visual or hearing deficit were included. The child’s health was assessed by a health questionnaire and by the survey International Study of Asthma and Allergies in Childhood. The spirometric test was performed on 3 different days, not exceeding the interval of 15 days between the first and last exams, following the guidelines of the American Thoracic Society. The absolute values and percentages of predicted values of forced vital capacity, forced volume in the first second and peak expiratory flow were analyzed. Statistical analysis was performed using the Shapiro-Wilk normality test followed by one-way analysis of variance or Friedman test and by Bonferroni’s post-hoc test for multiple parametric comparisons. Also, the intraclass correlation coefficient was applied to compare results on the same patients on the different tested days.

**Results:**

Twenty-two children were analyzed, with a mean age of 9.3±1.1 years. All the spirometric parameters showed a reduction in absolute value after the first exam, but without significant difference. Regarding reproducibility of spirometry results, the tests presented intraclass correlation coefficient >0.70.

**Conclusions:**

There was no change in the results of spirometry performed by healthy schoolchildren on three different days.

## RESUMO

**Objetivo:**

Analisar se há alteração no resultado do exame espirométrico executado por escolares saudáveis, quando realizado em dias diferentes.

**Métodos:**

Estudo transversal com escolares saudáveis com 7 a 12 anos, provenientes de escolas da Grande Florianópolis, Santa Catarina, Brasil. Foram incluídas crianças pré-púberes, não atletas, nascidas a termo, sem qualquer doença cardiorrespiratória, reumática, musculoesquelética, neurológica e déficits visuais ou auditivos. A higidez da criança foi avaliada por meio de um questionário de saúde e do questionário *International Study of Asthma and Allergies in Childhood*. O exame espirométrico foi realizado em 3 dias diferentes, não excedendo o intervalo de 15 dias entre o primeiro e o último exame, seguindo as diretrizes da *American Thoracic Society*. Foram analisados os valores absolutos e os percentuais dos valores preditos de capacidade vital forçada, volume forçado no primeiro segundo e pico de fluxo expiratório. A análise estatística foi realizada com o teste de normalidade de Shapiro-Wilk, seguida por análise de variância de uma via ou teste de Friedman e pelo teste *post-hoc* de Bonferroni para comparações múltiplas paramétricas. Foi aplicado o coeficiente de correlação intraclasse (ICC) para comparar os resultados dos mesmos pacientes entre os diferentes dias.

**Resultados:**

Vinte e duas crianças foram analisadas, com média de idade de 9,3±1,1 anos. Todos os parâmetros espirométricos apresentaram redução numérica no decorrer dos dias avaliados, no entanto, sem diferença significante. Na análise da reprodutibilidade da espirometria, os testes apresentaram coeficiente de correlação intraclasse >0,70.

**Conclusões:**

Não houve alteração do resultado da espirometria executada por escolares saudáveis em três dias distintos.

## INTRODUCTION

The evaluation of the pulmonary function has proven to a useful tool in children and adolescent developmental studies, as well as in the handling of chronic diseases.^1^ Therefore, spirometry is used as a valid and reliable instrument to assess respiratory integrity. To conduct the spirometry, the individual assessed needs to collaborate in order to perform the maneuvers requested in the spirometric test. In pediatrics, the need for special strategies from the technical team to conduct a successful exam is common.^2^ ^,^ ^3^

For the spirometry test to be valid, it needs to present acceptable and reproducible curves, without artifacts, according to the guidelines published by the American Thoracic Society, in 2005.^4^ The test is conducted in a standardized manner and is established for adults and preschoolers, basically following the same criteria for adults and children aged more than six years.^5^ ^,^ ^6^ However, children have technical difficulties to perform the maneuvers, often demonstrating lack of attention and difficulty to understand and cooperate during the examination,^7^ ^,^ ^8^ which can be justified by the age. The literature states that the main difficulty in this age group is to perform the maneuver of forced expiration, and maintaining the expiration during the period. Despite these difficulties its indication is frequent in the pediatric age group, since it involves the measurement of respiratory flows. Besides, the peak expiratory flow obtained in this test shows the cooperation of the patient.^9^ ^,^ ^10^

The interpretation of the spirometry helps to monitor respiratory conditions in childhood, and translates the scenario and the severity of pediatric diseases,^8^ as well as the response to different therapies.^11^ Besides, this instrument is used to confirm healthiness,^3^ growth control and pulmonary development^12^, and epidemiological studies.^13^ In this sense, field studies have been conducted using this tool in schools, rehabilitation centers, clinics and hospitals,^6^ ^,^ ^9^ ^,^ ^11^ favoring the evaluation of the pulmonary function in different populations,^14^ both in adults^15^ and in pediatrics.^16^

In the case of children, the performance in spirometry may be influenced by the understanding of the commands, by the reproduction of specific respiratory maneuvers, and by motor function.^17^ Therefore, its execution in different situations, environments and moments may influence the quality of the test. In this context, the objective of this study is to analyze if there is any change in the result of the spirometry, conducted with healthy school children, when the test is performed on different days.

## METHOD

A cross-sectional study was conducted from July 2014 to April 2015, with children aged between 7 and 12 years, attending public schools in Florianópolis, Santa Catarina, Brazil. This study was approved by the Ethics Committee of Universidade do Estado de Santa Catarina, report n. 708.446 (CAAE: 64 22676113.6.0000.0118).

It included healthy prepubertal children, non-athletes (not enrolled in a high-performance sports federation), born at term, without any cardiorespiratory, rheumatic, musculoskeletal, neurological disease nor visual or hearing deficit. This information was obtained through a health questionnaire (elaborated by the researchers), sent by the school the parents and/or tutors, together with the questionnaire *International Study of Asthma and Allergies in Childhood* (ISAAC). The exclusion criteria included the identification of asthma through the respective module in the ISAAC questionnaire, with cutoff point >5 for students aged between 6 and 9 years, and >6 for students aged between 10 and 14 years.^18^ The sample did not include obese school children (percentile equal to or higher than 97), according to the on-line calculator of the Ministry of Health with the Program Telessaúde Brasil,^19^ and those who were unable to execute any evaluation procedure. Data of ten children in a pilot study substantiated the sampling calculation. For that estimation, the variation of the spirometry parameter of FEV_1_ (forced expiratory volume in 1 second) was considered in the three moments analyzed. For a 12% difference to be detected in FEV_1_ and 85% test power, with 5% significance level, 18 students were considered to be sufficient to compose the final sample.^20^

Anthropometric data were collected: weight (digital scale, Ultra Slim W903 Wiso^®^, São José/SC, Brazil), height (portable stadiometer Sanny^®^, American Medical do Brasil Ltda., São Paulo/SP, Brazil), and body mass index (BMI).

For the analysis of the spirometry tests, the guidelines of the American Thoracic Society (ATS, 2005) were followed.^4^ The spirometer used was the EasyOne^®^ (Medizintechnik AG, Zurich), previously calibrated and connected with its software (notebook HP^®^, 2013, São Paulo/SP, Brazil). The absolute and percentage values of the predicted values were analyzed, according to Polgar and Weng,^21^ of forced vital capacity (FVC), forced volume in 1 second (FEV_1_), and peak expiratory flow (PEF). There was no bronchodilator test. Spirometry was conducted on different days; that is, the students had three tests (Esp1, Esp2 and Esp3), on three different days (Day 1, Day 2, and Day 3), not exceeding the maximum interval of 15 days between the first and the last test. Spirometries were performed by the same evaluator, who used the same incentive screen from the instrument’s software. The incentive was characterized by the simulation of blowing into a ball until it exploded. The verbal command was standardized and maintained during the tests. There was a minimum of three and a maximum of eight spirometry maneuvers, in each one of the three exams.

The Statistical Package for the Social Sciences (SPSS^®^, Chicago-IL, USA), version 20.0, was used to process the statistical analyses. The data were reported as measures of central tendency and dispersion. The data distribution was assessed using the Shapiro-Wilk test. To compare the spirometry data obtained on the three different days (Esp1 x Esp2 x Esp3), the repeated measures analysis of variance (ANOVA) of the Friedman test. Since there were significant differences, the Bonferroni post hoc test was used for parametric multiple comparisons. The reproducibility of spirometry exams was determined by the intraclass correlation coefficient (ICC) of two measures between Day 1 x Day 2, Day 2 x Day 3, and Day 1 x Day 3. A 5% significance level (*p*<0,05) was considered for all tests.

## RESULTS

Twenty-four children were assessed; however, 2 children were excluded for not meeting the acceptability and reproducibility criteria of the spirometry. Therefore, 22 children participated in this study (being 14 girls), with mean age of 9.3±1.1 years. These data are presented in Table 1.

**Table 1:** Anthropometric and spirometry characteristics of the sample.

	Mean±standard deviation	95% confidence interval
Age (months)	116.3±15.5	109.4-123.1
Height (m)	1.14±0.35	0.98-1.29
Weight (kg)	31.9±7.2	28.7-35.1
BMI (kg/m^2^)	17.1±2.1	16.2-18.0
SVC (%predito)	92.9±8.2	89.3-96.6
IC (L)	1.4±0.5	1.2-1.6
ERV (L)	0.6±0.6	0.3-0.8
FVC (L)	2.1±0.9	1.9-2.3
FVC (%predicted)	96.6±10.8	92.1-101.2
FEV_1_ (L)	1.9±0.8	1.7-2.1
FEV_1_ (%predicted)	92.5±10.9	87.7-97.4
VEF_1_/CVF	1.0±0.01	0.9-0.9
PEF (L/minute)	4.8±0.3	4.0-5.5
PEF (%predicted)	88.9±26.9	78.9-99.5
PEF_25-75_(L/minute)	2.3± 0.1	2.1-2.6
PEF_25-75_ (%predicted)	98.2±20.5	89.1-107.3

BMI: body mass index; SVC: slow vital capacity; IC: inspiratory capacity; ERV: expiratory reserve volume; FVC: forced vital capacity; FEV_1_: forced expiratory volume in 1 second; PEF: peak expiratory flow; PEF_25-75_: mean expiratory flow; L: litter.

**Figure 1:**
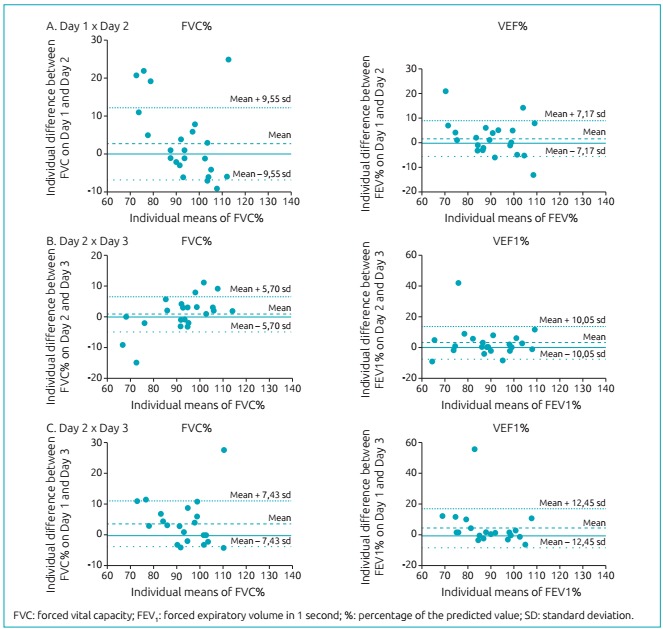
Distribution of percentage diferences of the predicted forced capacity and forced expiratory volume in 1 second between the moments of evaluation represented by the Bland-Altman graph.

All spirometry parameters presented a numerical reduction of their values throughout the assessed days. However, this difference was not significant (Table 2). When analyzing the reproducibility of the spirometry result obtained on each day of evaluation, the tests presented intraclass correlation coefficient >0.70, pointing to an excellent reproducibility (Table 3). In Figure 1, the distribution of the differences of FVC% and FEV_1%_ between the moments of evaluation is represented in the Bland-Altman graph.

**Table 2:** Distribution of spirometry data on the three analyzed moments (values shown in mean ±standard deviation).

	Day 1	Day 2	Day 3	p-value
FVC (L)	2.0±0.4	2.0±0.4	2.0±0.4	0.055^a^
FVC (%predicted)	95.6±2.2	92.8±3.0	91.9±2.3	0.090^a^
FEV_1_ (L)	1.8±0.8	1.8±0.9	1.7±0.9	0.089^a^
FEV_1_ (%predicted)	91.6±2.2	89.9±2.8	86.7±2.9	0.112^a^
PEF (L/minute)	2.2±6.7	1.7±1.2	2.0± 1.2	0.240^b^
PEF (%predicted)	98.3±6.2	98.3±5.2	99.0±5.6	0.610^b^
PEF_25-75_ (L/minute)	2.3±0.1	2.3±0.1	2.2±0.1	0.760^a^
PEF_25-75_ (%predicted)	92.1±3.4	91.7±4.7	90.6±5.3	0.950^a^

^z^ANOVA test; ^b^Friedman test; FVC: forced vital capacity; FEV_1_: forced expiratory volume in 1 second; PEF: peak expiratory flow; PEF_25-75_: mean expiratory flow.

**Table 3:** Distribution of data as to the intraclass correlation coefficient of spirometry parameters in the three days of the examination.

	Day 1 x Day 2 ICC (95%CI)	Day 2 x Day 3 ICC (95%CI)	Day 1 x Day 3 ICC (95%CI)
FVC (L)	0.96 (0.91-0.98)	0.98 (0.97-0.99)	0.97 (0.92-0.98)
FVC (%)	0.83 (0.60-0.93)	0.94 (0.87-0.97)	0.86 (0.68-0.94)
FEV_1_ (L/minute)	0.97 (0.94-0.99)	0.95 (0.88-0.97)	0.91 (0.79-0.96)
FEV_1_ (%predicted)	0.90 (0.76-0.95)	0.84 (0.61-0.93)	0.70 (0.29-0.86)
PEF (L/minute)	0.78 (0.49-0.91)	0.95 (0.90-0.98)	0.83 (0.60-0.93)
PEF (%predicted)	0.95 (0.90-0.98)	0.98 (0.95-0.99)	0.97 (0.94-0.99)
PEF_25-75_ (L/minute)	0.96 (0.91-0.98)	0.88 (0.72-0.95)	0.90 (0.76-0.95)
PEF_25-75_ (%predicted)	0.87 (0.68-0.94)	0.78 (0.47-0.91)	0.80 (0.53-0.91)

ICC: intraclass correlation coefficient; 95%CI: 95% confidence interval; FVC: forced vital capacity; FEV_1_: forced expiratory volume in 1 second; PEF: peak expiratory flow; PEF_25-75_: mean expiratory flow.

## DISCUSSION

This is the first Brazilian study that assesses the acceptability and the reproducibility of spirometry tests in healthy children, outside controlled laboratories. These tests have been widely used in field studies, once they assess pulmonary volumes and flows and are sensitive to identify possible alterations.^1^ However, the technical performance of students during the test is often complex, determining some level of concern in its conduction. In this sense, conducting the test in different moments seems to enhance relative risks, such as the learning effect, lack of motivation in the execution of maneuvers and very discrepant results. This study investigated spirometry evaluations conducted on different days, executed by healthy students. The results obtained on the three days of analysis were not different from each other, which suggests there may not be a learning effect and that this type of practice presents relative safety.

Studies in this sense have been developed especially with adults. In 1987, Larsson et al.^22^ assessed the spirometry of 21 individuals with chronic obstructive pulmonary disease, applied on three different days, in a two-week interval. The authors did not observe interference from the learning effect in this population, but identified a slight improvement in FVC values when the test was conducted in the afternoon.^22^ Spirometry tests conducted every two months, for a year, have also been objects of investigation. Even though the authors observed improvement in the results obtained with time, after investigating 1,135 tests they verified that the evolution was slow, late and had no statistical or clinical significance. The authors stated that the lack of knowledge and experience of the patients regarding the exam did not interfere in the results.^15^

There are few studies involving children in this field, probably because of the difficulty to conduct spirometry, inherent to the characteristics of this age group. It is known that children go through changes as individuals, resulting from their environment. They are also developing, which involves the improvement of different fields, such as cognition, language, motor and behavioral areas.^23^ ^,^ ^24^ Therefore, age is determinant in the execution of the test, since it is directly related with motor and cognitive acquisitions, enabling the better understanding of the commands, including the ones involved in spirometry maneuvers.^8^ ^,^ ^16^ The young age is the reason for some of the limitations of this test.^8^

In this population, one study put in practice the acceptability and reproducibility criteria published by the American Thoracic Statement/European Respiratory Statement (ATS/ERS), in 2005,^4^ with the objective of determining the influence of age on the ability to fulfill the spirometry criteria. It assessed children and adolescents aged 4 to 17 years, and 74% of the sample met the acceptability and reproducibility criteria of ATS.^4^ The success of the spirometry increased with age. The major difficulty attributed to the excluded tests was the lack of expiratory effort and the early glottis closure.^25^

In this study, children also met the acceptability and reproducibility criteria, and even if subject to the familiarization with the test and its repetition, because of the fact it was executed on three different days, the children’s performance did not change. A similar result was verified in a study conducted by França et al.,^1^ who assessed the reproducibility of the PEF in test-retest sessions, using the peak flow measurement device. Even though this equipment is different from the spirometer used in this investigation, both analyze expiratory effort, corroborating the result verified here.

The use of spirometry in field studies with students is common,^16^ however, some setbacks are frequent in these cases. In this study, it was observed that the transportation of equipment, the excessive number of stimuli from the data collection place and the environmental conditions may make the study difficult, besides the characteristics of the pediatric age group. However, the results obtained, even with a small sample, reinforce the credibility that a spirometry test may present when these elements are controlled and the technical criteria are assumed. It is suggested that other studies in this line, including children in different clinical situations and in other fields, such as rehabilitation centers, clinics and schools, be developed.

Finally, the spirometry result obtained on the three days of evaluation was not characterized by the learning effect nor by the familiarization with the equipment and the maneuvers involved. This finding indicates that the spirometry conducted in field studies with healthy school children, even if conducted on different days, does not compromise the results of the test.
